# Emergency Combination of Four Drugs for Bloodstream Infection Caused by Carbapenem-Resistant *Enterobacteriaceae* in Severe Agranulocytosis Patients with Hematologic Malignancies after Hematopoietic Stem Cell Transplantation

**DOI:** 10.1155/2020/9358426

**Published:** 2020-08-04

**Authors:** Xiaofan Li, Yaqun Hong, Xianling Chen, Ping Chen, Nainong Li

**Affiliations:** ^1^Hematopoietic Stem Cell Transplantation Center, Fujian Institute of Hematology, Fujian Provincial Key Laboratory on Hematology, Department of Hematology, Fujian Medical University Union Hospital, No. 29 Xinquan Street, Gulou District, Fuzhou 350001, China; ^2^The Graduate School of Fujian Medical University, No. 1 Xuefubei Street, Minhou County, Fuzhou 350108, China

## Abstract

Bloodstream infection (BSI) caused by multidrug-resistant (MDR) bacteria or extensively drug-resistant (XDR) bacteria is a global threat. However, an effective treatment regimen is still controversial and inadequate due to the rapid deterioration caused by the bacteria. In immunocompromised and neutropenic patients, MDR-BSI is an emergency, which causes treatment-related mortality. In this study, four agranulocytosis patients with hematologic malignancies after HSCT receiving treatment for carbapenem-resistant *Enterobacteriaceae*- (CRE-) BSI were included. Conventional treatment using two to three combined antibiotics was administered in the first and second patients. Combination treatment using four drugs, polymyxin B, high-dose tigecycline, fosfomycin, and double-dose carbapenem, was administered in the third and fourth patients. None of the patients receiving conventional treatment survived. Both patients receiving combination treatment using four drugs survived. Therefore, four-drug combination therapy may be needed in CRE-BSI patients who experienced severe agranulocytosis after HSCT. The efficacy of the four-drug combination treatment for CRE-BSI patients as well as the adverse effects need to be further studied.

## 1. Introduction

Bacterial infection is an important cause for mortality after hematopoietic stem cell transplantation (HSCT). Granulocytopenia due to the chemotherapy or radiotherapy in conditioning regimen leads to increased susceptibility for bacterial infections. Some can manifest as fatal bloodstream infections (BSI): high frequency of the multidrug-resistant (MDR) bacteria, even progressing to the extensively drug-resistant (XDR) bacteria and pandrug-resistant (PDR) bacteria. Multidrug-resistant (MDR) *Enterobacteriaceae*, including extended-spectrum *β*-lactamase (ESBL) bacteria and carbapenem-resistant *Enterobacteriaceae* (CRE), is a great threat to recipients in HSCT [1]. BSI caused by multidrug-resistant Gram-negative bacteria (MDR-GNB) after transplantation, mostly including *Escherichia coli* and *Klebsiella pneumoniae* [2], has shown a negative impact on long-term survival in transplantation [3]. Herein, we present four cases in which CRE was detected in neutropenic patients with hematologic malignancies and focus on the treatment of MDR bacteria.

## 2. Methods

### 2.1. Patients' Characteristics

From January 2019 to December 2019, a total of four consecutive severe agranulocytosis patients with hematologic malignancies after HSCT received antibiotic treatment for CRE-BSI. Two patients were male and two were female (see Table 1). The eligibility criteria included a diagnosis of CRE-BSI after HSCT, as well as absence of other active malignancies. No exclusions were made because of age, performance status, cardiac, hepatic, or renal function. This retrospective study was approved by the Ethics Committee of Fujian Medical University Union Hospital.

### 2.2. Supportive Care

Packed red blood cells and platelet transfusions were administered when patient hemoglobin level and platelet counts dropped below 60 g/L and 20 × 10^9^ cells/L, respectively. Five days after HSCT, 200 *μ*g/m^2^/day G-CSF was given subcutaneously until absolute neutrophil count (ANC) was higher than 1.5 × 10^9^ cells/L. Prophylactic antifungal therapy using posaconazole was included, while systemic antifungal therapy was empirically given for unexplained persistent fevers.

### 2.3. Laboratory Testing

MIC values of in vitro susceptibility tests with different drugs against CRE were determined in a multifunction laboratory study by the approved Committee for Clinical Laboratory Standards of Fujian Medical University Union Hospital, which included the evaluation of microdilution testing. Reference MIC values for the tested isolates were determined by agar dilution method with Mueller-Hinton agar according to CLSI guidelines. *E. coli* ATCC25922 was used as quality control strain. The results are shown in Table 2.

## 3. Results

**Figure 1:**
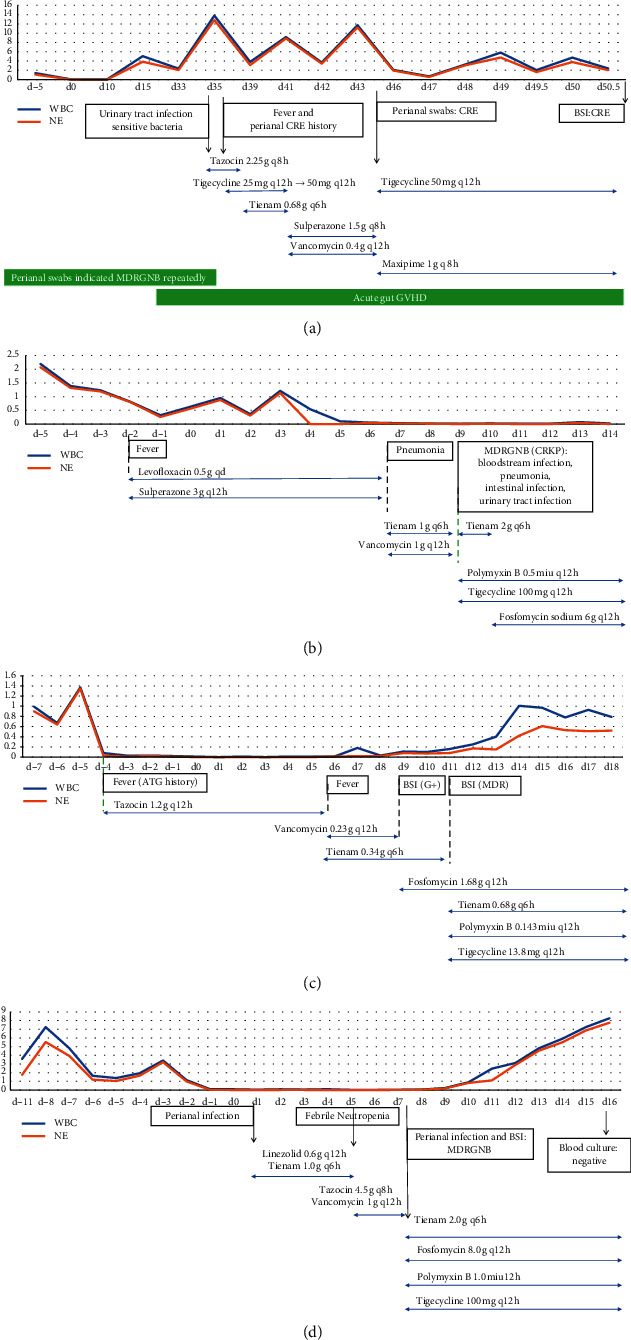
Blood cell counts, clinical cases, and treatment regimens of four cases. (a) Summary of blood cell counts, clinical cases, and treatment course of Ye. (b) Summary of blood cell counts, clinical case, and timing of antibiotic exposures of Zhang. (c) Summary of blood cell counts, clinical case, and timing of antibiotic exposures of Chen. (d) Summary of blood cell counts, clinical case, and timing of antibiotic exposures of Li.

Figure 1 shows the blood cell counts, clinical cases, time of antibiotic explore, and treatment courses of four cases. The first case is a six-year-old boy who was diagnosed with high-risk B-cell acute lymphocyte leukemia with detection of high triploid when he was 15 months old. Due to little response to chemotherapy, CAR-T was performed after the 20^th^ chemotherapy session. Subsequently, halpo-HSCT was utilized to treat the relapsed and refractory ALL. Acute gut GVHD manifesting recurrent diarrhea was diagnosed at day +20 after engraftment. Methylprednisolone, ciclosporin, and lucitanib were administered to treat acute gut GVHD. At day +35 posttransplantation, tazocin, 2.25 g, q8h, was used due to a probable intestinal infection manifesting with fever and diarrhoea accompanied with acute gut GVHD. Given the history of CRE in perianal swabs culture repeatedly, tigecycline was added subsequently according to antibiotic susceptibility. However, the fever was not controlled and increased inflammatory markers were detected in blood. Thus, tazocin was changed into tienam, and the dose of tigecycline was doubled. Three days after treating with this strategy, clinical symptoms did not disappear and more severe diarrhoea developed. Thus, an adjustment using sulperazone, 1.5 g, q8h, and vancomycin, 0.4 g, q12h, was carried out. Recurrent fever and diarrhea continued, while the latest result of perianal swab culture indicated CRE detected, and tigecycline was sensitive solely. Tigecycline and maxipime were given at day +45 posttransplantation. The patient died from acute gut GVHD with persisting blood stools 51 days after transplantation.

The second case is a 25-year-old female diagnosed with hemophagocytic syndrome with recurrent fever. Salvage haploidentical transplantation was performed. After a high-dose chemotherapy treatment, the patient suffered from granulocytopenia. At day +3 posttransplantation, due to recurrent fever accompanied with chills and an increase of inflammatory markers, including IL-6 and procalcitonin, levofloxacin, 0.5 g, qd, sulperazone, 3 g, q12h, and indometacin were used. However, the patient continued to be feverous recurrently and subsequently developed intestinal infection manifesting diarrhea at day +7. Thereby, tienam, 1 g, q6h, and vancomycin, 1 g, q12h, were administrated to replace levofloxacin and sulperazone. Further lab test, including urinary tract, perianal swab, and blood bacterial culture, indicated a progression to systematic XDR bacterial infection, with drug susceptibility showing that almost all the antibiotics are resistant, except colistin and tigecycline. Consequently, combination therapy using polymyxin B, tigecycline, and fosfomycin were administrated. However, the patient died from CRE-BSI at day +14 posttransplantation.

The third case is a 29-month-old boy diagnosed with acute monocytic leukemia with abnormal karyotype. During hospitalization, antibiotic was first used at day +4 due to febrile neutropenia, which might be caused by the administration of ATG or infection. Six days after HSCT, the patient showed an increase of inflammatory markers. Thus, tazocin was escalated to tienam, 0.34 g, q6h, and vancomycin. At the same time, anal fissure was considered because of another chief complaint of perianal pain, which might be the origin of CRE-BSI detected at day +11. An emergency combination therapy with four drugs, including polymyxin B, tigecycline, fosfomycin, and double-dose carbapenem, was administrated immediately. Finally, the following results of blood culture showed no bacteria detected. The patient is still alive and has regular followups.

The fourth case is a 50-year-old woman with multiple myeloma who underwent an autologous HSCT. Four days before HSCT, several pustules were found in her crissum. Considering her history of perianal abscess and septic shock, perianal infection was considered, and mupirocin topical ointment was applied. Due to the aggressive presentation of perianal infection at day +1 posttransplantation accompanying granulocytopenia, linezolid, 0.6 g, q12h, and tienam, 1.0 g, q6h, were added. There was no improvement by day +5 posttransplantation with a fever presenting, so tazocin and vancomycin were administered. Lab results of blood and perianal swabs cultures indicated BSI and perianal infection with carbapenem-resistant *Klebsiella pneumoniae* (CRKP). An emergency combination therapy with four drugs, including polymyxin B, tigecycline, fosfomycin, and double-dose carbapenem, was administered immediately, after which CRE was cleared. The patient is still alive and has regular followups.

## 4. Discussion

Immunosuppression patients with BSI are considered an emergency event in clinical practice. Classification of bacteria is broadly based on bacterial properties, such as staining, oxygen tolerance, or morphology. Using Gram stain, bacteria can be categorized into Gram-positive and Gram-negative bacteria [4]. Based on the characteristics of Gram-negative bacteria, the antibacterial action of drugs is determined. For example, the interaction between colistin and LPS can enhance the permeability of the cell envelope and lead to an antimicrobial action [5]. Also, the binding of *β*-lactams with penicillin-binding proteins (PBPs) deactivates enzymes, which play an important role in the assembly or morphogenesis of the bacterial cell wall [6, 7]. However, CRE resist most antibiotics used in clinical practice which make it more difficult to deal with [8].

Immunosuppression and prolonged neutropenia are the most common conditions in patients undergoing HSCT, which are risk factors for infection. The mortality related to BSI caused by CRE is 51% in adult neutropenic patients [9]. We presented four cases of BSI caused by CRE. Only two patients using combination therapy with four drugs survived. The first case could not receive an effective treatment before the final result of blood test was reported, and using tigecycline and maxipime seemed to have a little effect on treating infection of CRE though a MIC <0.5 was detected. It is critical that the median day from the onset of bacteremia until death was about four days [9]. Therefore, for such high-risk patients, an emergency combination therapy should be considered early. The second patient was treated with combination therapy too, which included polymyxin, tigecycline, and fosfomycin. However, the patient died. We highly suspect that carbapenem might be required when treating CRE-BSI in immunosuppression patients. Thus, we used an emergency combination therapy with four drugs to treat patients subsequently, and clinical successes were achieved.

In the “Turin bundle,” the combination therapy using colistin 9 MU, then 4.5 MU, q12h + tigecycline, 100 mg, q12h + meropenem, 2 g, q8h. was recommended for severe sepsis/septic shock caused by CPKP carriers [10]. Though meropenem was not advised when MIC was higher than 8–16 mg/L in a study by Bassetti et al. [11], an enhanced efficacy of double-dose carbapenem therapy compared with carbapenem alone was observed in both in vitro chemostat and in vivo murine model [12]. Also, the double-dose carbapenem regimen demonstrated a synergistic effect in a neutropenic patient suffering from BSI caused by XDR-CPKP after allo-HSCT whose blood test indicated MIC >32 of carbapenem and clinical successes were achieved in 3/7 (43%) patients with BSI caused by CRKP [13, 14]. Eventually, double-dose tienam was administrated in our combination therapy. Due to no new or alternative antibacterial drugs available and drawbacks such as the risk of emergent resistance and suboptimal efficacy, combination therapy with some agents that remain active in vitro, including colistin/polymyxin B and tigecycline, should also be considered [15]. Colistin is effective against XDR carbapenemase-producing microorganisms both in vitro and in vivo, and a combination therapy is necessary to treat bacteremia in the critically ill patients [16]. Another “old” drug, fosfomycin, was suggested in combination therapy. A synergy was observed when fosfomycin was combined with meropenem or colistin [17]. A synergistic effect could also be found in the combination of colistin with meropenem and tigecycline [18]. It is also important to note that the 100 mg tigecycline twice daily showed a higher efficacy in patients with hospital-acquired pneumonia compared with lower doses of tigecycline [19]. Considering these drug interactive findings, our empirical therapy combining polymyxin B, high-dose tigecycline, fosfomycin, and double-dose carbapenem was administered, and immunocompromised and neutropenic patients in cases four and three survived BSI caused by CRE.

## 5. Risk Factors for CRE, BSI, and Mortality

Apart from the treatment regimen for BSI, many risk factors for HSCT mortality should also be considered. All patients reported here had PICC before transplantation and no underlying disease, yet many characteristics of patients are heterogeneous. The retrospective case report includes two children and two adults who were diagnosed as ALL, HLH, AML, and MM, respectively, and showed different clinical histories. Source of stem cells varied that were collected from mobilized peripheral blood of HLA-haploidentical related donor and single cord blood of unrelated donor or single cord blood of unrelated donor or autologous mobilized peripheral blood. All patients undergoing allo-HSCT received intensified myeloablative conditioning. The patient undergoing auto-HSCT received myeloablative conditioning using CBV regimen. Perianal mucosal damage was observed in two of them. The previously mentioned factors might be related to the course and severity of infection. Considering the patients' clinical infection condition, combination therapy could be considered as an emergency treatment.

It is worth noting that colonization was significantly related to MDR bacterial infections in HSCT, which commonly results in bacteremia eventually, and what is also noteworthy is the high rate of patients colonized by at least one MDR organism [20–23]. For pre-HSCT rectal swab screening in the four cases, CRE was detected repeatedly in the first case and sensitive *Enterococcus faecalis* was observed in the third case, while the results of cases three and four were negative. Rectal swabs are positivity correlated with the subsequent BSI caused by MDRGNB. CRKP is a predictive indicator of subsequent infection in immunocompromised patients [24]. Though a study by Forcina et al. showed no significance between a pretransplant MDR-GNB colonization and the overall survival, transplant-related mortality, or infection-related mortality in HSCT [25], most studies indicated that colonization with MDR organisms is an important risk factor for nonrelapse mortality of HSCT [20, 22, 26]. Preengraftment Gram-negative bacteremia is associated with the rise of mortality rate at four months after auto-HSCT and allo-HSCT [26]. Therefore, an evaluation protocol and timing for the execution of the rectal swab is necessary. A well-defined monitoring strategy has not been standardized. We executed rectal swab before transplantation, and the rectal swab results of these four cases are shown in Table 1. Regular detection was performed every two weeks unless related symptoms and signs, such as perianal mucosal damage or abnormal stool, were found. Additionally, it is still controversial whether there is a significant difference between colonization and outcome of HSCT and whether therapies should be given when perianal or rectal swabs culture result indicates a MDR infection without any other evidence of infection before transplantation. Moreover, the therapy strategies for colonization after transplantation are inconclusive. A significant survival difference was described between patients who cleared the MDR organisms and those with persistent MDR organisms, but interestingly, it is the length of antibiotic therapy that is a potential risk factor for MDR organism persistence after HSCT [27]. It is worth noting that the first case also developed GVHD. Acute gastrointestinal GVHD is associated with the rise of enteric bacterial BSI in pediatric allo-HSCT recipients [28]. The further multicenter study of BSI showed that enteric bacterial BSI after the onset of acute gastrointestinal GVHD caused a nearly fivefold increase in transplantation-related mortality [29]. Acute GVHD grades II–IV are an independent predictive indicator of subsequent first BSI, and both acute GVHD grades II–IV and BSI predict death within 100 days after HSCT [30].

Another possible factor for causing MDR is other previous antibiotic therapies, such as fluoroquinolone, which is administrated in the second case, and the third-generation cephalosporins [31–33]. However, some studies described that prophylactic treatment with levofloxacin showed a lower rate of bacteremia by contrast [34, 35]. The use of fluoroquinolones in patients with cancer and neutropenia is still controversial. It is also important to note that the pediatric patient in the first case developed acute gut GVHD after HSCT, which is maybe associated with MDR. There is a new and diversified pattern of accumulation of antimicrobial resistance genes in pediatric patients who develop acute GVHD after HSCT, including multidrug resistance [36]. It is noteworthy that, due to a prolonged bacterial culture, the results of the blood bacterial culture of the first case came out after death, which increased the difficulty of treatment and was a risk factor for mortality. A study by Satlin et al. also described a critical data that the 30-day mortality of patients who were infected with CRE in blood stream but treated with antimicrobial agents before receiving final antimicrobial susceptibility testing were 9/9 [9]. Another risk factor for mortality may be the antimicrobial susceptibility. Multiple factors impact on the incidence and outcome of BSI, and both early prevention and combination therapy are important.

## Figures and Tables

**Table 1:** Characteristics of patients.

	Case 1	Case 2	Case 3	Case 4
Name	Ye	Zhang	Chen	Li

Age (year)	6	25	2	50

Gender	Male	Female	Male	Female

Weight (kg)	21	41	12	65.9

Diagnosis	Relapsed and refractory ALL (CR)	Hemophagocytic syndrome (NR)	Acute monocytic leukemia (CR)	Multiple myeloma

Underlying disease	None	None	None	None

Treatment before HSCT	Multiple chemotherapy; CAR-T	Multiple chemotherapy	Multiple chemotherapy	Multiple chemotherapy

EBMT score	3	4	2	—

Type of HSCT	Allo-HSCT	Allo-HSCT	UCBT	Auto-HSCT

Source of stem cells and donor	Peripheral blood of HLA-haploidentical related donor (5/10); single cord blood of unrelated donor (7/10)	Peripheral blood of HLA-haploidentical related donor (5/10); single cord blood of unrelated donor (5/10)	Single cord blood of unrelated donor (7/10)	Peripheral blood

Intensity of conditioning	Intensified myeloablative conditioning	Intensified myeloablative conditioning	Intensified myeloablative conditioning	Myeloablative conditioning (CBV)

Had PICC device	Yes	Yes	Yes	Yes

Pre-HSCT rectal swab	MDRGNB repeatedly (CRE). Day –5: CRE	Negative	Day –11: *Enterococcus faecalis* (sensitive)	Negative

Post-HSCT rectal swab	Day +4: CRE Day +42: CRE Day +46: CRE	Day +10: CRE	Day +7: CRE	Day +1: CRKp Day +9: CRKp

Duration of neutropenia	—	From day –2 to day +14 (died before engraftment)	From day –4 to day +15	From day –1 to day +10

Development of GVHD	Grade IV	None	None	None

MDR bacteria	CRE (*Escherichia coli*)	CRKP (*Klebsiella pneumoniae*)	CRE (*Escherichia coli*)	CRKP (*Klebsiella pneumoniae*)

Treatment	Combination therapy with two drugs	Combination therapy with three drugs	Combination therapy with four drugs	Combination therapy with four drugs

Outcome	Died from acute GVHD and infection	Died from infection at day +14	Improved	Improved

Reduction in the number of transplanted T cells in haplo-HSCT	Yes	Yes	Yes	Yes

Perianal mucosal damage before BSI	None	None	Perianal mucosal damage	Perianal mucosal damage

ALL: acute lymphocytic leukemia; UCBT: umbilical cord blood stem cell transplantation; haplo-HSCT: haploidentical hematopoietic stem cell transplantation; allo-HSCT: allogeneic hematopoietic stem cell transplantation; auto-HSCT: autologous hematopoietic stem cell transplantation; CRE: carbapenem-resistant *Enterobacteriaceae*; GVHD: graft versus host disease.

**Table 2:** Antibiotic susceptibility of MRDB detected (MIC value).

Antibiotics	Case 1:Ye	Case 2: Zhang	Case 3: Chen	Case 4: Li
Amikacin	≥64	≥32	≤2	≥64
Aztreonam	≥64	≥64	≥64	≥64
Ciprofloxacin	≥4	≥4	≥4	≥4
Doxycycline	—	≥16	≥16	≥16
Cefepime	—	≥32	≥32	≥32
Imipenem	≥64	≥16	8	≥16
Levofloxacin	≥8	≥8	≥8	≥8
Meropenem	—	≥16	8	≥16
Minocycline	—	≥16	4	≥16
Trimethoprim/sulfamethoxazole	≥320	≥320	≥320	≥320
Ceftazidime	—	≥64	≥64	≥64
Tobramycin	≥16	≥16	≥16	≥16
Piperacillin-tazobactam	≥128	≥128	≥128	≥128
Cefoperazone/sulbactam	—	≥64	≥64	≥64
Colistin	—	≤0.5	≤0.5	≤0.5
Tigecycline	≤0.5	4	≤0.5	2

## Data Availability

The data used to support the findings of this study are available from the corresponding author upon reasonable request.

## Additional Points

BSI caused by MDR or XDR bacteria in immunocompromised and neutropenic patients after HSCT is a global threat. In a study by Satlin et al., the 30-day mortalities are 4/10, 6/16, or 4/8 in single, two, or three Gram-negative agent(s), respectively [9]. However, effective treatment regimen is still controversial and inadequate. Our emergency therapy combining polymyxin B, high-dose tigecycline, fosfomycin, and double-dose carbapenem saved two patients in our center, but verification using in vitro, in vivo, and well-designed clinical studies may be needed in the future. Moreover, the adverse effect is unknown and a long-term followup is needed. It is noteworthy that the use of colistin extensively or inadequately may cause resistance of colistin among *K. pneumoniae* isolates [37]. However, strong combination therapy should be considered in immunocompromised and neutropenic patients because of the rapid deterioration and high mortality. New drugs, such as zavicefta, may provide a better strategy to deal with the dilemma [38]. Given the heterogeneity, more data and analysis of related cases are needed.
